# Editorial Notes

**Published:** 1943

**Authors:** 


### Editorial Notes

**Mr. Winston Churchill and Standish House.**

STANDISH HOUSE, now the Gloucestershire Tuberculosis Sanatorium, has the distinction of having belonged to the family from whom the Prime Minister and Chancellor of our University, derives his first christian name. A recent note in *Country Life* pointed out that Sir Winston Churchill, father of the great Duke of Marlborough, obtained his christian name from the surname of his mother. She was Sara, daughter and heiress of Sir Henry Winston of Standish, Gloucestershire, and she married John Churchill in 1618.

**"The Practitioner."**

WE offer our congratulations and good wishes to *The Practitioner* on celebrating its seventy-fifth anniversary. *The Practitioner* was founded in 1868 under the joint editorship of Francis Edmund Anstie, then Senior Assistant Physician, Westminster Hospital, and Henry Lawson, Assistant Physician, St. Mary's Hospital. Anstie is a name well known in Bristol. The family were tobacco manufacturers at Devizes, and the business still flourishes, managed by the family as a branch of the Imperial Tobacco Company. In the June, 1943, issue of *The Practitioner*, Sir Humphry Rolleston has contributed an article on the history of the Journal, in which memoirs of the former editors are collected. The longest and most interesting memoir is that of Anstie, whose ideals have inspired the Journal from its inception to the present time, namely, to provide an essentially practical publication with special emphasis on the latest information on the treatment of disease. This emphasis on what Sir Thomas Watson described as "the greatest gap in the Science of Medicine," namely, our knowledge of therapeutics, continues to be the reason for the success of *The Practitioner* under its present controlling Editor, Sir Humphry Rolleston, and the Joint Editor, Dr. Alan Moncrieff.

**New Regulations for the F.R.C.S.**

THE Council of the Royal College of Surgeons of England has revised the regulations of the Fellowship and at last the diploma of F.R.C.S. can be regarded as a qualification equivalent to a Mastership of Surgery. The Council is to be congratulated on having adopted the view that the F.R.C.S. should be confined to those who practise the higher branches of surgery, just like the Fellowships of the other two English Colleges. The *primary* examination beginning on November 29th, 1943, will be the last to be conducted under the present regulations.

New regulations have been approved by the Council and will become effective as from the end of 1943.

These regulations embody the following changes :—

(i.) The *primary* examination cannot be taken by undergraduates, but will be open only to Members of the College, or to Graduates in medicine and surgery of the Universities and Medical Colleges recognized by the Council for the purpose, who are able to comply with the conditions of the regulations.

(ii.) The subjects of the primary examination will be :— (a) *Anatomy* (*including Normal Histology*), *and* (b) *Applied Physiology and the Principles of Pathology.* A synopsis indicating the general scope and spirit of the examination in *applied physiology and the principles of pathology* is published in the new regulations.

With regard to the *final* examination, no candidate will be admissible without producing evidence of having been engaged in the acquirement of professional knowledge for not less than two years subsequent to the date of having obtained the Membership of the College or some other recognized qualification, *vide* (i.) above.

The dates of the examinations have been re-arranged, so that it will be possible for candidates who pass the *primary* examination to proceed immediately to the *final* examination, if they are eligible. During 1944 the examinations will begin on the following dates :—

*Primary* examination—April 24th and October 23rd.
*Final* examination—May 4th and November 2nd..

Copies of the new regulations, and full particulars may be obtained, post free, from the Director of Examinations, Examination Hall, Queen Square, London, W.C. 1.

